# A Unifying Mechanistic Model of Selective Attention in Spiking Neurons

**DOI:** 10.1371/journal.pcbi.1003577

**Published:** 2014-06-12

**Authors:** Bruce Bobier, Terrence C. Stewart, Chris Eliasmith

**Affiliations:** Centre for Theoretical Neuroscience, University of Waterloo, Waterloo, Ontario, Canada; Philipps-University Marburg, Germany

## Abstract

Visuospatial attention produces myriad effects on the activity and selectivity of cortical neurons. Spiking neuron models capable of reproducing a wide variety of these effects remain elusive. We present a model called the Attentional Routing Circuit (ARC) that provides a mechanistic description of selective attentional processing in cortex. The model is described mathematically and implemented at the level of individual spiking neurons, with the computations for performing selective attentional processing being mapped to specific neuron types and laminar circuitry. The model is used to simulate three studies of attention in macaque, and is shown to quantitatively match several observed forms of attentional modulation. Specifically, ARC demonstrates that with shifts of spatial attention, neurons may exhibit shifting and shrinking of receptive fields; increases in responses without changes in selectivity for non-spatial features (i.e. response gain), and; that the effect on contrast-response functions is better explained as a response-gain effect than as contrast-gain. Unlike past models, ARC embodies a single mechanism that unifies the above forms of attentional modulation, is consistent with a wide array of available data, and makes several specific and quantifiable predictions.

## Author Summary

At a given moment, a tremendous amount of visual information falls on the retinae, far more than the brain is capable of processing. By directing attention to a spatial location, stimuli at that position can be selectively processed, while irrelevant information from non-attended locations can be largely ignored. We present a detailed model that describes the mechanisms by which visual spatial attention may be implemented in the brain. Using this model, we simulated three previous studies of spatial attention in primates, and analysed the simulation data using the same methods as in the original experiments. Across these simulations, and without altering model parameters, our model produces results that are statistically indistinguishable from those recorded in primates. Unlike previous work, our model provides greater biological detail of how the brain performs selective visual processing, while also accurately demonstrating numerous forms of selective attention. Our results suggest that these seemingly different forms of attentional effects may result from a single mechanism for selectively processing attended stimuli.

## Introduction

Several decades of physiology, imaging and psychophysics research on attention have generated an enormous amount of data describing myriad forms of attentional influence [1]–[5]. A similar breadth of theoretical models have been proposed that attempt to explain these effects in varying amounts of detail [3], [6]–[10]. However, models that simultaneously are able to reproduce attentional effects from multiple studies, and that provide a neurally detailed mechanism are rare. Consequently, there remains a need for neurally detailed mechanistic models, especially those which provide experimentally testable predictions. The work presented here aims to identify principles that underlie selective attentional processing in cortex, and that are consistent with a wide variety of neuroanatomical and neurophysiological constraints. Specifically, we describe a functional mechanism for attentional routing in a large-scale hierarchical model, and demonstrate the biological plausibility of the model by presenting a spiking neuron implementation that accounts for five forms of attentional effect. Specifically, we demonstrate that the model exhibits shrinking and shifting, but not amplitude changes of spatial receptive field (RF) profiles; an increase of tuning curve gain without sharpening; and response gain effects in neuronal contrast-response functions.

Several attempts have been made to explain multiple and seemingly disparate forms of attentional modulation in a single model, but each has significant limitations. The model presented here, the Attentional Routing Circuit (ARC) overcomes limitations of past models by providing a unified explanation of this set of attentional effects, by being implemented in spiking neurons, by defining the relationship of attentional modulation across multiple cortical areas, by quantitatively comparing the model to physiological data, and by making several detailed testable predictions. The ARC is fully implemented in spiking neurons and specifies how top-down feedback signals can be used to perform selective routing of attended visual stimuli in cortex. The model defines this process at multiple levels of abstraction, from a large scale network comprising multiple hierarchical cortical areas, to the laminar microcircuitry of cortical columns, and to the attentional modulation of single cells.

## Materials and Methods

**Figure 1:**
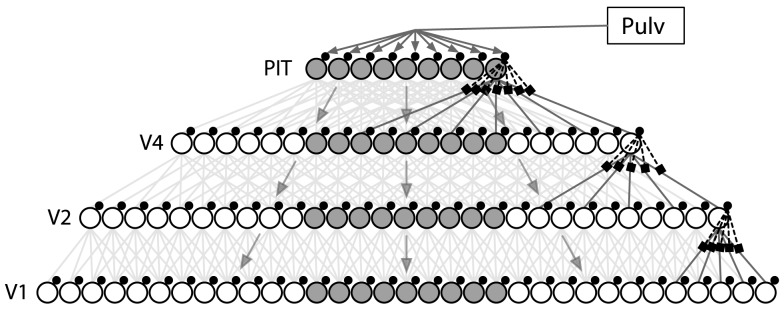
General architecture of the ARC. Each level has a columnar and retinotopic organization, where columns (large circles) are composed of visually responsive neurons (not individually depicted) and control neurons (small circles). Large filled circles indicate columns representing an example attentional target. Each column receives feedforward visual signals (gray lines) and a local attentional control signal from control neurons (dashed lines), and these signals interact nonlinearly in the terminal dendrites of pyramidal cells (square boxes). The application of this architecture to ventral stream processing is shown here. Global control signals from pulvinar are projected to PIT and then fed back to control neurons in lower levels. Connectivity is highlighted for the rightmost columns only, although other columns in each level have similar connectivity.

We begin by describing, at the local level, a prototypical cortical circuit for attentional routing in the ARC. The basic organizational unit used in the ARC is the cortical column, which we use to refer to an assembly of neurons spanning the six laminae, and located within close spatial proximity. Our usage of the cortical column is for organizational convenience, and does not rely on such columns having specific boundaries (c.f. [11], [12]). Rather, functionality can vary smoothly across columns having more gradual horizontal boundaries. The model assumes that the mechanisms for selective routing are the same for all columns in all levels of the visual hierarchy. Thus, at the global or network level, the control of selective attentional routing is governed by all columns using a common cortical algorithm where, in aggregate, the individual columns all contribute to routing the target, as specified by the global control signal. The global layout of these columns for the ventral visual pathway is depicted in Figure 1.

**Figure 2:**
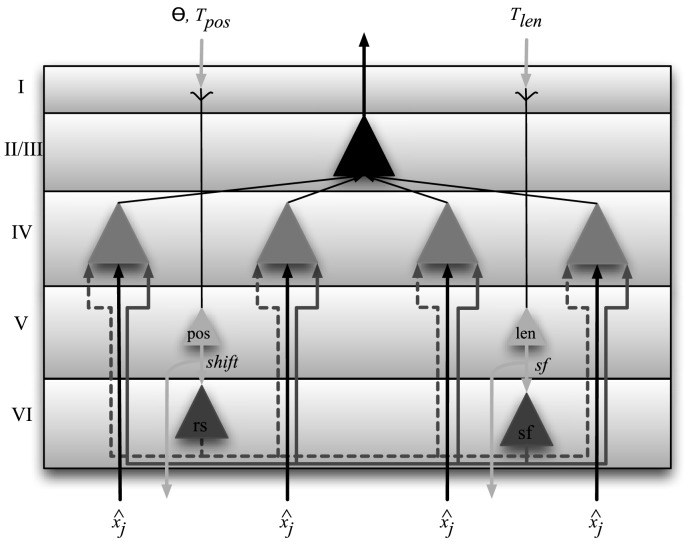
Laminar circuitry of attentional control in the ARC for a single column. Global attention signals that include the size (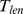), position (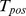) and center of mass (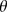) are fed back from the next higher cortical level to layer-I where they ramify on apical dendrites of layer-V cells (see Equations 1–5). Layer-V neurons relay this signal to the next lower area with collaterals projecting to control neurons in layer-VI of that column where a sampling factor (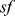) and relative shift (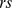) are computed (see Equations 1 and 4 respectively). These signals, along with feedforward visual signals carrying image information 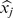 are received by layer-IV pyramidal cells where the routing function is computed in the dendrites and multiplied with 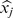. Cells in layer-II/III pool the activity of multiple layer-IV neurons and project the gated signal to the next higher level. See text for additional details.

The ARC divides the laminar anatomical distinctions along functional lines as well [13], [14]. The ARC proposes that neurons in layers-V and -VI compute local control signals, while neurons in layer-IV are involved in the selective gating of inputs based on control signals from the deep layers, and neurons in layer-II/III process the gated visual signals. The proposed physical and functional organization of neurons in each cortical layer for a single column is shown in Figure 2. Although other plausible variations to this mapping may be found, this arrangement is consistent with the most salient and well documented inter-laminar connections [15]–[18], and requires minimal control neurons and wiring. Ascribing a particular function to neurons in a cortical layer places constraints on the possible computations that may be performed due to the anatomical connectivity and neuronal density of that layer. At a minimum, it permits assessing the plausibility of the proposed functions being performed in actual cortical circuits, by ensuring that the number of neurons required by the model does not obviously exceed the number found in the corresponding cortical area.

The ARC seeks to minimize the neuronal requirements for routing in terms of the number of neurons and the length of their axonal and dendritic processes, by having neural populations that compute local control signals for all visually responsive neurons in their column. The proposed arrangement permits the model to be scaled to have significantly more neurons in each column that encode a variety of visual features and receive feedforward inputs from a spatially broad set of columns, while requiring a reasonably small number of control calculations.

### Computing Global Feedback

We now describe the network and methods for performing selective attentional routing in cortex. We then briefly define the methods for computing connection strengths for nonlinear functions using the Neural Engineering Framework (NEF). We will present the computations and connections of ARC by describing and directly mapping them to their cortical counterparts. A central assumption of ARC that is shared with several other models [6], [10], [14], [19], is that the mechanisms by which attentional routing is performed in cortex are consistent across areas in the ventral and dorsal visual hierarchies. For example, although cells in V4 and MT are selective for different stimuli, they share many structural and morphological properties [12], [20], [21], and ARC proposes that the mechanisms and computations used for attentional routing of attended stimuli are consistent between these areas.

Within each column, we distinguish two functional classes of neurons: visually responsive neurons and control neurons. Layer-II/III contains visually responsive neurons that encode visual stimuli, while visually responsive cells in layer-IV having nonlinear dendrites gate feedforward visual signals based on local control signals (see Figure 2). Layers-V and VI contain control neurons that compute and relay local control signals.

All neurons are modelled as LIF neurons [22], with refractory periods of 2 ms, membrane time constants of 20 ms, and maximum firing rates drawn from a uniform distribution in the range [90, 120] Hz. For simplicity, neurons are responsive to a single dimension for contrast, although including responsiveness to additional feature dimensions is straightforward and does not affect the model's principles. However, it will increase the number of neurons needed to reproduce the simulations described here. The following equations define the computations for performing selective routing between V4 and PIT, although identical equations define all transformations between every other set of adjacent levels in the circuit.

Selective routing in the ARC begins when pulvinar projects a global control signal that coarsely encodes the size and position of the attentional target (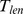 and 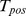) to cortical control neurons in the top level of the hierarchy (PIT; see Figure 2). These pulvinar-cortical projections utilize only a fraction of pulvinar connectivity and computational capacity, and thus do not preclude its involvement in other processes. The global control signal is defined in terms of the number of V1 columns that the target spans and its position with respect to the fovea. These global control signals (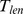 and 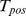) specify the size and position of the target for the entire network. Local control signals, 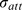 and 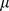 are computed based on the global control signals, and specify the width and centre of the local Gaussian routing function for individual columns. That is, global control signals are specified in retinotopic coordinates and describe the spatial properties of the entire object being attended, whereas the local control signals are defined in terms of their RF position, and guide the routing of a local portion of the attended object.

Feedback projections from higher cortical areas terminate in layer-I upon the apical dendrites of layer-V intrinsically bursting (IB) pyramidal cells that receive the global control signal 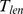. Such neurons have large cell bodies and long apical dendrites that spread widely in layer-I, providing them with access to the broadly distributed signals from the area above [16], [18]. These neurons relay 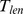 to dendrites in layer-I of the preceding area in the hierarchy [15] and to the dendrites of nearby layer-VI pyramidal cells in the same column [17].

The control neurons in layer-V of PIT first determine the size of the target's representation in V4. If the target's size in V1 (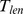) is larger than the number of columns in V4 (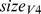), then its representation will span all columns in V4. Otherwise, the target's size in V1 will be maintained in V4. Typically the number of V4 columns encoding the target will exceed the total number of PIT columns (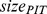), requiring its representation to be reduced between V4 and PIT. This is accomplished by having each PIT column selectively process information from V4 columns separated by a "sampling factor'' 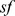: 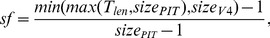
(1)where 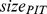 and 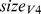 are constant. The synaptic weights between the layer-V and layer-VI neurons approximate the function for computing 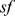 (Equation 1). In short, these control neurons determine an appropriate sampling factor for fitting the target in V1 into PIT, via V4. The sampling factor, 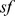 attempts to minimize the loss of information by having as many columns encode information from the target as possible, but not more than encode the target in V1 (i.e. the target size, 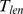).

Other layer-V neurons with dendritic arbours in layer-I receive feedback projections that encode 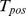 and 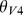. Here, 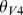 represents the centre of mass of the target's representation in V4. These layer-V neurons have similar laminar connectivity to those receiving 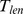, but instead use the global control signal 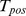 to compute the relative shift (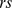) of the target's representation from the preceding level. Due to cortical magnification, wherein a significantly greater proportion of neurons respond to foveal stimuli than to peripheral stimuli [23], this calculation seeks to have the target's representation (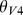) as close to the centre of the level as possible: 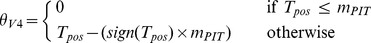
(2)

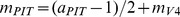
(3)where 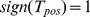 if the target is in the right hemifield (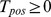) and -1 otherwise, 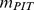 represents the maximum shift that can occur for columns in PIT, and 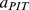 is the number of afferent V4 columns providing input to each PIT column. For a given network architecture, 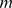 is constant in each level, and is defined as the sum of half of the RF size for cells in that column (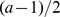) and the maximum shift of the level below. Shifting the target's representation to the centre of each level takes advantage of the Gaussian sensitivity profile of visual cortical neurons [6], which is particularly important for the large receptive fields in higher cortical areas.

Having computed the position of the target's representation in V4 (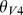) and assuming an object-centred reference frame in PIT (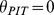), the number of columns by which the centre of the target's representation is shifted between V4 and PIT is the difference between the centre of the target's representation in V4 and in PIT: 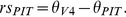
(4)


We note that the computations thus far are not unique to particular columns in an area, but rather are the same values throughout the level. The values for the sampling factor (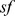), target position (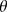) and relative shift from the level below (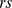) are the same in all columns in the level. Although we have described these calculations as being performed within each column, the strong lateral connectivity of layer-V neurons [18] may allow the calculations to be performed in a distributed and less redundant manner, thereby requiring fewer total neurons to be involved.

### Default and Selective Routing

Both 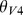 and the global control signals 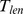 and 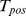 are fed back from the layer-V cells in PIT to layer-I in V4. Depending on the target's size and position however, only some V4 columns will switch from their default routing state to a selective routing state in which they process the target. To determine the routing state for neurons in a given column, each V4 column receiving the feedback signals computes: 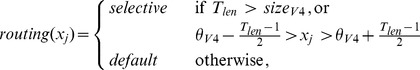
(5)where 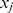 is the 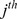 input column in the previous level (with 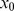 being at the fovea and negative values being in the left hemifield) and 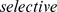 indicates that the column will perform selective routing. V4 columns that are not selectively routing the target remain in their default routing state, and the global attention signals are blocked before they can influence the activity of that column or be fed back to V2. In short, a given V4 column will perform selective routing if the target spans more V1 columns (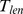) than the number of V4 columns (i.e. all columns in V4 will encode the target), or if its position is within 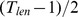 columns of the target's centre in V4 (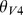).

### Computing Local Control Signals

The control neurons involved in computing Equations 1 and 4 project 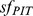 and 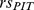 to visually responsive layer-IV neurons in their column. The layer-IV neurons are modelled using a 2-layer neuron model with nonlinear dendrites [25], where each layer-IV neuron receives inputs from a single column in the preceding level. Pyramidal cells with nonlinear dendrites have been found in neocortex and hippocampus [26]–[29], and based on the morphological and electrochemical similarities of those cells and pyramidal cells in visual cortex, it is proposed that such neurons may also be found in visual cortex. Neurons with dendritic nonlinearities offer a significant computational advantage over classical point neuron models, since, in addition to the standard axosomatic nonlinearity, they may also compute a second nonlinear function of their inputs in the dendrites, thereby offering processing capabilities similar to a two-layer artificial neural network [25]. As a result, the number of neurons needed in each level is much less than if linear dendrites are assumed. ARC's dendritic integration of feedback signals is highly consistent with the hypothesized backpropagation activated calcium (BAC) spike firing. BAC firing allows individual cortical pyramidal neurons process two individual information streams independently, then combine the processed information to significantly increase their computational power [30].

To reduce the computational demands of control neurons, the ARC attempts to minimize the number of highly nonlinear functions, as such functions tend to require more neurons in order to be computed accurately. Further, in computing local control signals, the linearly separable parts of the routing function are distributed across different groups of neurons, allowing each ensemble to compute a more tractable function. Combining these components of the routing function together in the dendrites yields the same result, while significantly reducing the neuronal requirements. To quantify the efficiency improvements of such an approach, we found that dendritic nonlinearities can be exploited to perform selective routing, with a decrease in the number of cells needed by a factor of 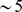 as compared with a linear dendrite model (see [31] for details).

The synaptic weights of the layer-IV nonlinear dendritic subunits serve to approximate the routing function and determine the RF location from which visual signals should be selectively processed: 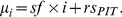
(6)


**Figure 3:**
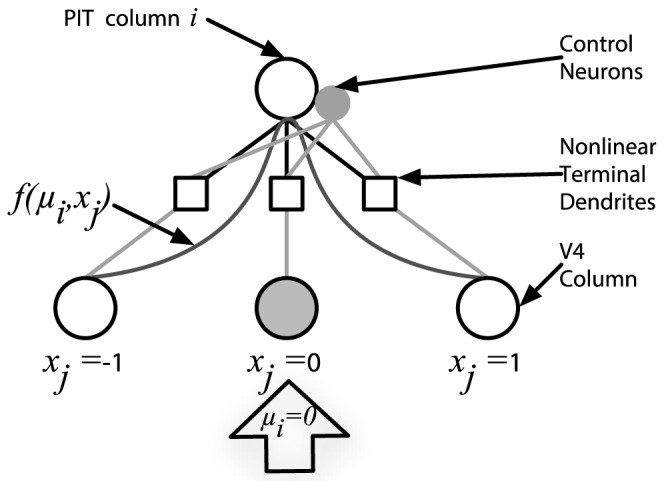
Selective routing for a single PIT column. The Gaussian routing function 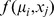 is centred on input column 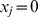 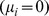, and control neurons in the PIT column project the local control signal to the dendritic subunits of layer-IV neurons in that column. The gain of visual signals from each column is scaled by the corresponding value produced by the routing function.

This value 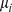 specifies the centre of the Gaussian-shaped routing function defined over the RF of neurons in column 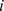 in PIT and serves to modulate the gain of visual signals from input columns throughout its RF according to the routing function: 
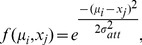
(7)


where, as before, 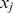 is a constant representing the spatial position of the 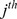 input column in V4 (Figure 3). That is, 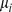 specifies the location within the cell's RF from which it should selectively route information without attenuation, while stimuli elsewhere in the RF are attenuated proportional to their distance from 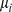.

If the number of V4 columns encoding the target exceeds the total number of PIT columns, 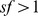, then some V4 columns that encode the target could be skipped over, and their visual information may not be processed in PIT. To minimize the loss of information from the target in such cases, the width of the routing function, 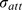, is adjusted to cover such columns by dividing 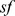 by the full width at half maximum: 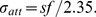
(8)


With the routing function computed in the dendrites, the product of 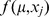 and 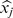 is then computed using the second nonlinearity, where 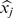 is the signal carried by neurons in column 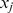. The gated visual signals are projected to neurons in layer-II/III, which pool gated visual signals from other layer-IV neurons within their column. Cells in layer-II/III are thus primarily encoding part of the target and can project this as feedforward visual information to layer-IV cells in the next higher cortical area [17].

The entire ARC model (Figure 1) uses these computations between each level in an identical manner. However, the underlying neurons implementing these computations are randomly chosen from distributions reflecting the variety of neurophysiological properties found in visual cortex. Consequently, the responses of the specific cells in a model vary in a biologically plausible manner. In addition, neurons and neural ensembles in each area are responding to and encoding different attributes of sensory information. However, we emphasize that in simulating different areas, (e.g. V4 vs MT), no changes are required to the mechanisms used to perform selective routing, but only to the distributions from which the cells preferred stimuli are drawn (e.g. orientation vs motion direction). The simulations in this article employ 1400 spiking neurons per column to compute all of the described functions. As noted earlier, including cells selective to more complex stimuli does not increase the number of control neurons needed. As a result, the model is expected to scale well, which we will examine in more detail in future work.

### Neural Engineering Framework

The Neural Engineering Framework (NEF) [32] is used to implement the signal processing described in Figure 2 in spiking neurons. This framework has been used to model a wide variety of neural circuits, including what is currently the largest available functional brain model [33]. The NEF allows the mathematical description of a dynamical system to be translated to a spiking neuron model [34]–[36]. Details of this procedure and the framework can be found in [31], [32], [37]. Here, we describe how the NEF can be used to analytically derive the connection strengths between populations that approximate the functions necessary for attentional routing.

The first principle of the Neural Engineering Framework (NEF) [32] is that the activity of a neural population "represents'' information in an underlying vector space. Specifically, the neural representation of the vector space is defined by the combination of nonlinear encoding (e.g. neuron tuning curves and spiking) and weighted linear decoding over neural populations and time. The tuning curve determines the cell's encoding of the vector space and may be expressed as 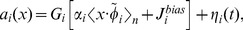
(9)where 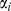 is gain, 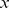 is the vector space, 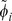 is the preferred direction vector, 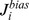 is the bias term corresponding to background activity, 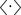 is the inner product of the 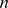 dimensional vectors, 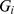 is the output nonlinearity that transforms somatic current to spiking activity, and 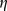 is a noise term. In all of the simulations presented in this article, 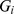 represents the standard leaky integrate-and-fire (LIF) nonlinearity [22], although other neuron models may be used. The LIF neuron model is used as it provides a suitable trade-off between biological realism and computational efficiency. The particular choice of neuron model that is used (e.g. adapting-LIF, Hodgkin-Huxley, etc.), does not affect the principles of the NEF. Nor does it influence the neural coding in the model, as the NEF does not depend on how the spikes are generated, but only on the statistics of the spike generation [32, pp. 89]. Thus, future work may replace the LIF neurons presently used with a more biologically detailed neuron model to investigate issues related to spike timing, synchrony, and oscillations.

The output activity 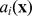 represents the neural response in terms of firing patterns, and this value is greatest when the input 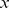 is aligned with the neuron's preferred direction vector, 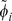. With spiking neurons, the activity of neuron 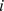 is defined as a sum of action potentials: 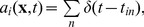
(10)where 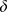 is an impulse representing a spike, 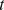 represents time, and 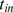 is the time of the 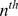 spike produced by neuron 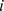.

Decoding in the NEF is a linear operation, determined as a weighted sum of the neuronal activity: 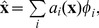
(11)where 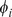 is the decoding vector and 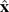 is the decoded estimate of 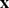. To analytically derive the decoding vectors 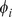, we minimize the error between the decoded estimate and the actual value of 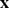 as:
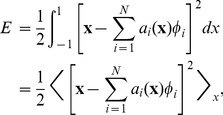
(12)where 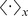 is the integral over 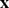. Solving for the decoders 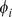 gives:
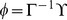
(13)

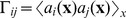
(14)

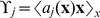
(15)


The temporal decoding of the neural spikes is mapped to the post-synaptic temporal responses of receiving cells as captured by the post-synaptic current (PSC). For present purposes, the PSC is modeled as a decaying exponential with a time constant determined by the receptors at the relevant synapse, i.e., 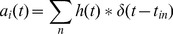
(16)

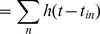
(17)where 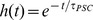. Consequently, the overall population temporal decoder for NEF representation is: 
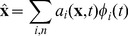
(18)where 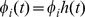, and 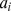 is defined by Equation 10.

Using this characterization of representation, we can directly compute the connection weights of a communication channel between presynaptic neuron 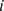 in population A and postsynaptic neuron 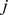 in population B by computing the product of their encoding and decoding vectors, scaled by a gain term: 
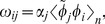
(19)where 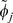 is the encoding vector of neuron 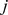 specifying its preferred direction, 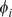 is the decoding vector of neuron 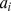 and 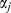 is the gain. These weights are found by substituting the decoding equation (Equation 11) into the encoding equation for population B (Equation 9). Thus, the activity of post synaptic neuron 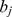 is found as: 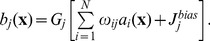
(20)


A communication channel is equivalent to computing the identity function between populations A and B. This same method can be extended to computing an arbitrary function 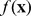 between these populations, as described by the second principle of the NEF. Specifically, the same methods for deriving the decoders 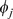 for a communication channel can be used to find the decoders 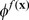 by substituting 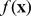 for 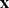 in Equation 12. Substituting these decoders into Equation 19 then provides the weights for approximating the function 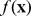 in the computation that occurs between populations A and B. The third principle of the NEF, describing the implementation of arbitrary dynamical systems in spiking circuits, is not used in this work, and so not described here.

## Results

To assess the plausibility and accuracy of the model, we use it to directly simulate electrophysiological studies. After showing that the model captures the experimental data, we then provide several experimentally testable predictions that, when tested, will either either validate the model, or highlight where it is wrong and needs to be adjusted.

Each of the three studies simulated here describes seemingly different forms of attentional modulation, although the ARC is able to reproduce each effect using the same neural mechanism for selective routing. That each of these effects can be demonstrated using the same routing mechanisms in a biologically detailed model, and without adjusting parameters, suggests that the ostensibly different forms of modulation may in fact be a consequence of a common underlying routing principle.

### Attentional Effects on Receptive Field Profiles

**Figure 4:**
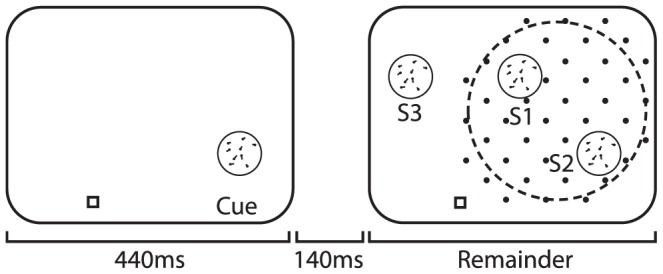
Experimental method used in Womelsdorf et al. [3] . See text for details.

Womelsdorf et al. [3] measured receptive field (RF) profiles in macaque area MT while two random dot pattern (RDP) stimuli were presented in the RF (S1 and S2), and one RDP stimulus was outside the RF (S3). While maintaining fixation, one of the three RDP stimuli was cued, and the animal covertly attended the cued stimulus to detect a transient change in motion direction (see Figure 4). For each attentional condition, higher contrast probe stimuli were presented in and around the RF and responses to the probes were fit with a Gaussian to construct a RF profile. They found that attending to a RF target resulted in the Gaussian RF peak shifting toward the target, while the width shrunk, without any significant change in peak activity.

**Figure 5:**
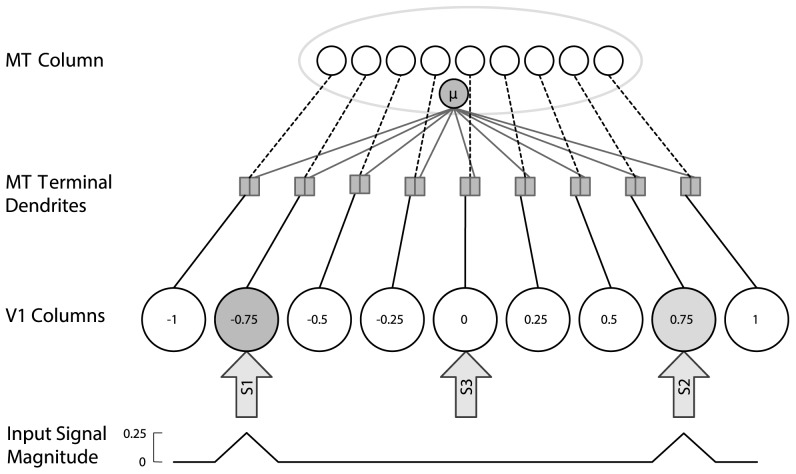
ARC model used for simulations. A single MT column contains 450 visually responsive layer-IV neurons (small white circles), and control neurons (small gray circle) that project a local control signal (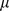) to enable selective attentional processing. Nine V1 columns constitute the receptive field of the MT column and provide feedforward visual signals. The spatial position of V1 columns is indicated inside the V1 columns and the magnitude of the visual signals encoded by each column is shown at the bottom of the figure. Attentional targets for simulations of [3] are shown as arrows (S1, S2, S3), with S3 corresponding to the attend-out/default routing condition.

The experiment was simulated using the ARC as shown in Figure 5. This implementation can be thought of as part of the large-scale ARC (Figure 1), demonstrating how selective routing may be performed between two adjacent cortical areas. We described the ARC in the context of the ventral stream to indicate its application to a multi-level hierarchy, and here we apply it to a single level in the dorsal stream to address the available empirical evidence. As noted earlier, in spatial attention tasks, the mechanisms for routing in ARC are the same in MT and V4, although the preferred stimuli of cells in those areas are drawn from different distributions. The two level network used to simulate this and all subsequent experiments is composed of a single MT column containing 100 layer-VI control neurons, 100 layer-II/III neurons, and 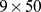 layer-IV pyramidal neurons each having 30 nonlinear dendritic subunits. The RF of cells in the MT column covers nine V1 columns containing 100 layer-II/III neurons that provide feedforward visual signals. Connection strengths between V1 and MT are set with a Gaussian profile with 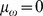 and 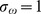 (see Materials and Methods).

Layer-II/III MT neurons have maximum firing rates drawn from a uniform distribution in the range [90, 120] Hz, and respond more strongly to higher contrast stimuli. 100 cortical control neurons in MT also project to the terminal dendrites of layer-IV MT cells, a local control signal that indicates the target's position within the RF (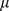). In the dendrites of the layer-IV MT pyramidal cells, the feedforward visual signals (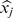) and local control signal (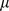) interact according to Equation 7. Layer-IV neurons then project the gated visual signals to 100 layer-II/III pyramidal cells, from which the spiking activity was recorded and used for analysis. For space reasons, we limit our analysis to only the layer-II/III neurons, and will investigate cells in other layers in future work.

The input to the MT column are visual stimuli from V1 and a top-down spatial attentional signal presented to the control neurons (capturing the cuing of attention in the experiment). The attentional signal specifies only the target's centre of mass and width. Two non-preferred reference stimuli with a contrast of 0.25 from the range [0, 1] were positioned at identical eccentricity in the RF, with all other inputs set to zero. Preferred probe stimuli with a contrast of 0.5 were then presented at each of the seven remaining V1 columns while the activity of all 100 layer-II/III MT neurons was recorded. The local control signal focused attention on the left stimulus (S1; 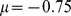) or right stimulus (S2; 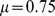; Figure 5). For the "attend-out'' condition, the attentional control signal for the MT column is set to 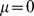 (S3). In [3], the peak of the RF in the attend-out condition occurs at a point that is approximately equidistant between S1 and S2. Directing attention to S3 corresponds to neurons operating in a default routing state where the width of the routing function (Equation 7) is set to 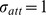, while for the attend-in conditions, 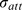 is set to 0.75. The value for 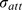 in the attend-in condition was not selected by parameter fitting, but rather as being a number smaller than in the default routing/attend-out conditions. To further underscore that the model does not depend on particular parameter values, these same parameter values are used for the simulations of all three studies presented here.

Following the same analysis methods used in [3], the average response to each of the seven probes was calculated with the baseline activity subtracted, and these data were fit by a Gaussian. All analysis of effects are identical to those in [3].

For statistical power and to demonstrate the robustness of the general architecture, 100 different simulated monkeys were used. Here, we use the term "simulated monkey'' to refer to a single instantiation of the ARC model in the NEF. This term allows us to distinguish the ARC in general, from a single *instance* of the ARC that has particular neuronal ensembles and connectivity. Parameters describing neuron tuning curves were randomly selected for each simulated monkey according to the distributions described in Materials and Methods; consequently, the connection weights for each simulated monkey are different. The data were bootstrapped (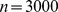), and used to compute 95% confidence intervals for each test.

Across the 100 simulated monkeys, on average, 30.34 of 100 neurons were discarded using the same criterion as in [3]. Of the remaining neurons, the median 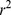 value of the fit was 96.43%. Following Womelsdorf et al. [3], neurons having an 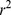 value from the Gaussian fit greater than the median for all three attentional conditions are referred to as "selected pairs.'' When all neuron pairs are examined, we refer to this as the entire sample.

The first attentional effect examined is a change in RF amplitude, calculated as (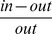), where 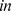 and 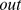 are the gain terms from the RF fits of the attend-in and attend-out conditions respectively. The distribution of activity changes for individual neurons was highly non-normal for both the selected pairs and entire sample (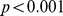, Jarque-Berra test), so the data were aggregated by taking the mean amplitude changes from each simulated monkey. The change in amplitude for the entire sample was non-significant (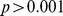, one sample t-test, 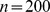), with an mean change of −0.343% (CI = [−1.485%, 0.797%]). The change for the selected pairs was also non-significant (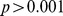, one sample t-test, 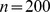), with a mean of −0.583% (CI = [−1.733%, 0.577%]). Both values from the simulations overlap with the 95% confidence intervals of the experimental data, and cannot be statistically distinguished. Consequently, the model shows the same lack of change in RF amplitude.

We then measured RF shift as (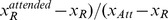), where 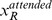 is the mean of the Gaussian fit with attention directed to a RF stimulus, 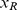 is the mean of the Gaussian fit in the attend-out condition, and 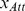 is the position of the local control signal, 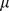. As with the amplitude change, the data were not normally distributed (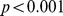, Jarque-Berra test). Using the aggregated mean change in RF position from the entire sample showed a statistically significant shift (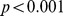, one sample t-test, 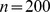), with a mean of 33.231% (95% CI  =  [32.864%, 33.620%]). The RFs of the selected pairs also significantly shifted (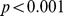, one sample t-test, 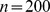), with a mean of 33.407% (95% CI  =  [33.037%, 33.776%]). These results, as well as those obtained using individual neurons rather than the aggregated means, are within the 95% confidence interval of the original experimental data (data not shown).

Changes in RF size were then quantified by measuring the change in width of the Gaussian fits between the attend-out (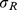) and attend-in (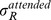) conditions, as 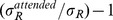. Using the aggregated mean of the entire sample from each simulated monkey, the RF size significantly decreased (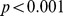), on average by −13.567% (CI = [−14.425%, −12.694%]). For the selected pairs, there was also a significant decrease in RF size (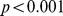), with a mean change of −16.193% (CI = [−17.190%, −15.183%]). The analysis of using all individual neurons are consistent with those obtained with analysis of the aggregate data. As with the previous two attentional effects, the simulation data cannot be statistically distinguished from the experimental data.

**Figure 6:**
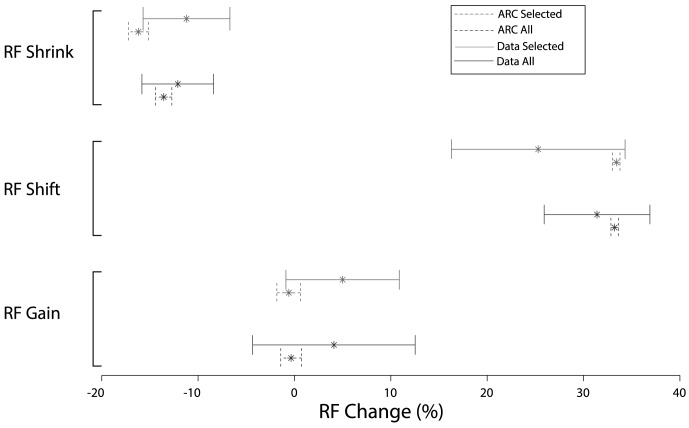
Summary of attentional effects (mean and 95% CI) for 100 simulated monkeys (dashed lines) and results reported by [3] (solid lines). Bars on the top for each effect are selected pairs and the lower below are from the entire sample.

Figure 6 summarizes the changes in RF properties from the simulation data (dashed lines) and experimental data (solid lines), for both the selected pairs (upper bars) and entire sample (lower bars). Asterisks indicate the mean change with 95% confidence intervals. Across these three measurements, there is no statistical difference between the simulation data and the experimental data.

These results show that the ARC's mechanism for selectively processing visual stimuli produces neural activity modulation that is statistically indistinguishable from the results recorded in macaque by Womelsdorf et al. [3]. Specifically, directing spatial attention to a RF target can reduce the width and shift the position of receptive fields without altering the amplitude.

Several studies have reported that attention can alter the tuning and selectivity of cortical neurons as well [2], [8], [38]–[40], and in the following section, we investigate this phenomenon by examining the effects of spatial attention on neuronal tuning curves.

### Attentional Effects on Feature Tuning

Treue and Martinez-Trujillo [2] recorded from macaque area MT while the monkeys covertly attended one of two RF stimuli or a stimulus placed at the fixation point. Stimuli were two RDPs, one moving in the cell's anti-preferred direction (Pattern A), and the other moving in one of 12 directions (Pattern B). On each trial, either stimulus was designated the attentional target, and the monkey had to detect a change in target speed or direction. Tuning curves were constructed for each cell by fitting the responses to the 12 stimulus directions in each condition with a Gaussian.

They found that attending the variable motion stimulus produced an increase in response gain, whereas attending the anti-preferred stimulus produced a reduction in gain, with an intermediate response when attending the neutral stimulus. Significantly, they also demonstrated that the width of these curves did not change between attentional conditions, but rather the responses were similarly multiplicatively scaled for all motion directions. Whereas Womelsdorf et al. [3] examined the impact of spatial attention on spatial RF profiles, Treue and Martinez-Trujillo [2] examined the impact of spatial attention on tuning curves.

To simulate the study, 100 simulated monkeys were used, with each having the same model configuration, but with neuronal and connection weight properties varying randomly, as in the previous simulations. As before, for the attend-in conditions, the local control signals were centred on the stimuli, while in the neutral condition, the control signal was 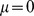 to reflect the default routing state. As in the previous simulations, 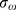 and 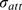 were again set to 1.

Following [2], we measured the change in gain and width when attention is directed to Pattern B as an attentional index (AI): 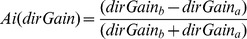
(21)

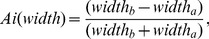
(22)where 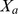 and 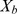 indicate attention being directed to Pattern A or B respectively.

Across the simulated monkeys and attentional conditions, the mean 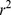 value of fitting the tuning curves with a Gaussian was 0.9847.

As with the previous simulations, the mean change across the population of cells in each simulated monkey were aggregated. When attention was directed to the variable direction stimulus (Pattern B), there was a significant increase in the attentional index for gain (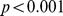), with a mean of 0.1564 (95% CI  =  [0.1529, 0.1597], corresponding to an increase of 137.39% (95% CI  =  [136.41%, 138.32%]). Consistent results were obtained when individual neurons were analysed (data not shown).

Further, when attending Pattern B, the width of the tuning curve did not significantly change (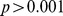), with an average attentional index of −0.00018 (95% CI  =  [−0.0033, 0.0031]), corresponding to a change of 100.2% (95% CI  =  [99.58%, 100.86%]).

From the histograms in the original article, (Figure 4 in [2]), confidence intervals of the mean can be estimated by taking the number of cells in each histogram bin, and assigning to these points, the value corresponding to the centre of the histogram bin. This estimated data set was then bootstrapped (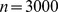) and used to estimate confidence intervals. For directional gain, this yields an average AI of 0.2125 (95% CI  =  [0.1518, 0.2768], and an average AI for width of 0.0357 (CI  =  [−0.0179, 0.0821]).

**Figure 7:**
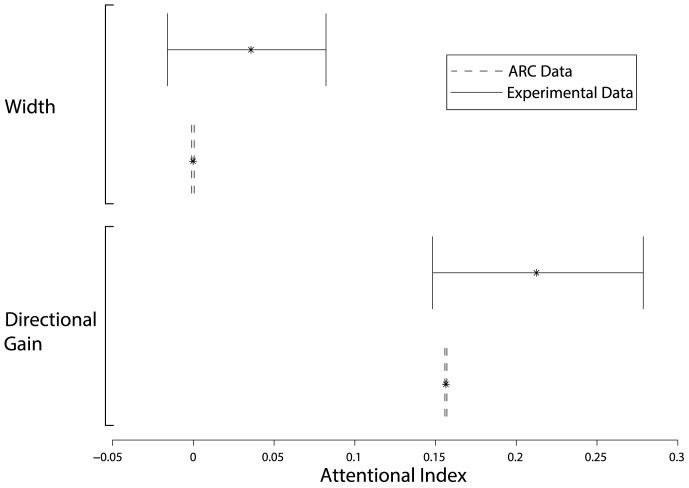
Summary of attentional effects on neural selectivity reported by [2] (solid lines) and from simulations using the ARC (dashed lines). The 95% confidence intervals of the simulation and experimental data overlap, with both showing a significant increase in gain, but no change in width.

Figure 7 shows that the ARC captures the attentional effects reported in [2], with a consistent change in gain, but no change in width. That is, our simulation data are statistically indistinguishable from the original experimental data.

These results distinguish the ARC from previous models. Specifically, the biased competition [8] and normalization model [10] predict that attending to the variable motion stimulus will produce a sharpening of the neuron's orientation tuning curve. However, the experimental data demonstrate that the tuning curve width is unaffected by directing spatial attention to different RF targets, and is discussed further in Discussion.

The ARC predicts that visuospatial attention affects the processing of information based on its retinal or RF location, independent of the feature information from a given location. Note that although the spatial width of the RF may change with shifts of attention, as was shown in the previous simulations, the range of features to which the neurons respond do not. In short, although shifts of spatial attention alter the spatial profile of RFs, spatial attention does not alter neuronal selectivity for other features.

This prediction of the ARC, that spatial attention similarly modulates all features, is further explored in the following section where we examine the effects of spatial attention on contrast selectivity.

### Attentional Effects on Contrast-Response Functions

Lee and Maunsell [5] recorded from macaque area MT while two Gabor stimuli with equal contrasts were presented in the RF. One stimulus moved in the cell's preferred direction, and the other in a non-preferred direction. The task was to detect the appearance of a Gabor at the cued location having a slightly faster drift speed. They found that the effect of spatial attention on contrast response curves was significantly better explained by a response gain effect than by contrast gain. Of the numerous studies examining attentional effects on contrast-response functions [40]–[42], the experiments of Lee and Maunsell were selected as they directly compare both response gain and contrast gain, and avoid possible analytic confounds of previous studies (see [5] for details).

To simulate this experiment in the ARC, we used the same network as before, with the following three extensions. First, as the feature of interest is contrast, for which neurons have specific sensitivity [13], [43], this property was included by having the 200 layer-II/III MT neurons begin responding above baseline to contrast values drawn from a uniform distribution in the range [0, 10%]. This extension does not alter the results of the previous two simulations, as it does not influence the information being processed or encoded by the cells, but only the manner in which the information is encoded.

Second, two sources of variability were introduced. Noise was added by injecting the soma of the layer-II/III MT neurons with a random amount of current drawn from a uniform distribution in the range [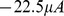, 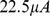] at each time step. As well, to increase the variability introduced by neurons below MT, V1 signals were scaled by a factor randomly chosen from a uniform distribution in the range [75%, 125%]. Although this has the potential to introduce a considerable amount of noise in the input signals, as shown below, its effects on neural activity are less apparent when the activity is averaged across 36 repetitions. This second extension also does not alter the previous results, as it only serves to increase the response variability across trials, without influencing the mechanism by which visual signals are gated or processed.

Third, to capture the high contrast sensitivity of MT neurons, the gain of layer-II/III neurons in V1 and MT was reduced to produce saturation for contrasts greater than 30%. Such neurons are able to well represent signals having values within the expected contrast range, although values beyond this range result in saturation of spiking activity, as well as of the estimate of the signals represented by that activity. As with the first extension, this does not affect the previous simulations, as the visual signals are selectively routed based on their spatial position and not the information carried by the neural activity. Thus, the extensions do not alter the ARC's mechanism for selective routing, but only affect the visual signals that are presented to the model; all signals are selectively routed in the same manner, based on the spatial position of the signals and not their content.

Following the same methods as in [5], the mean responses across the 36 repetitions for each attentional condition and contrast value were first fit with a hyperbolic ratio function [43]: 
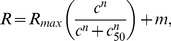
(23)where 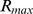 is the maximum firing rate, 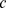 is contrast, 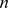 is the steepness of the function, and 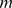 is the baseline activity.

**Figure 8:**
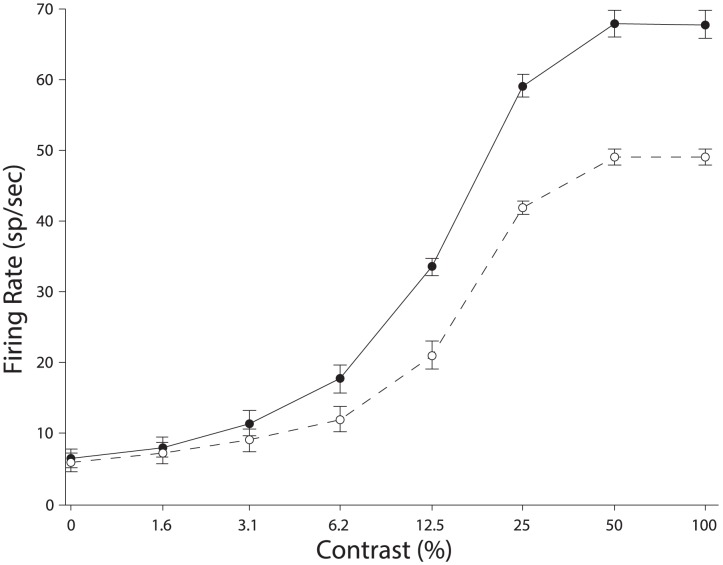
Population average contrast-response functions for 200 model neurons. Vertical scaling of responses with attention to the preferred stimulus show a predominately response gain effect (See Fig. 2C in [5]). The solid and dashed lines are the best fitting function when attention was directed to the preferred and non-preferred stimulus respectively. Error bars are SE values.

Figure 8 shows the average activity of the entire population when fit with the hyperbolic ratio function (Equation 23). For all 200 neurons, the median variance explained was 99.7% with attention directed to the preferred stimulus and 99.8% for attending to the non-preferred stimulus.

Following [5], to assess whether the data were better explained by a response gain or contrast gain, the mean responses from each attentional condition were fit with modified contrast response functions that model either a pure response gain or pure contrast gain. The response gain model incorporates an additional term, 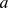, that scales the contrast-response function: 
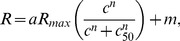
(24)and the contrast gain model is given by: 
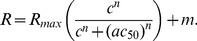
(25)


For both models, 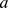 was fixed at 1 when attention is directed to the non-preferred stimulus and varied freely when attention was directed to the preferred stimulus.

The partial correlation was then calculated using the correlation between the data and each model's fit, and Fisher's 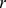-to-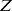 transformation was applied to each partial correlation coefficient to produce a 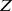 score. The transformed values were then divided by the standard error (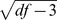), where 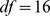 is the number of data points, with 8 contrast values in the two attentional conditions.

**Figure 9:**
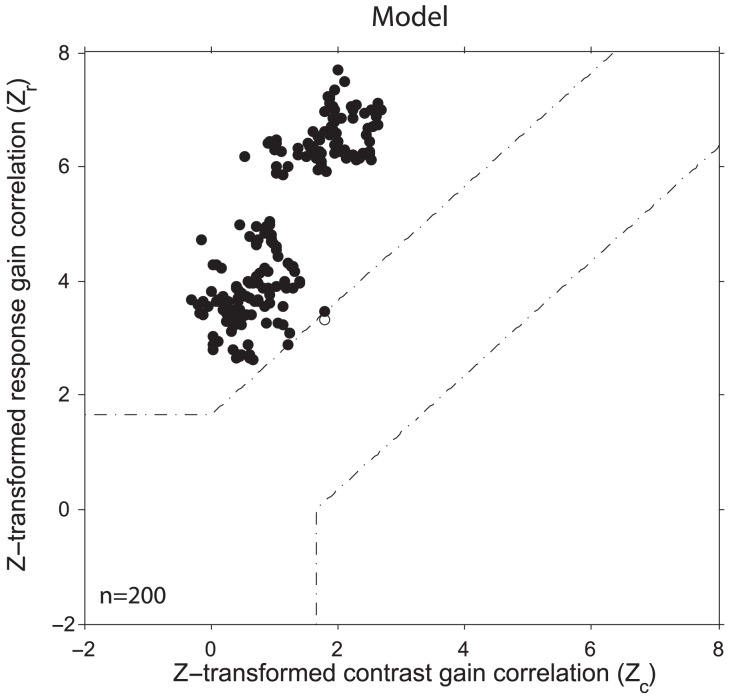
Z-transformed partial correlations between simulation data and curve fitting. Black circles are neurons having fits that are significantly better described by the response gain model. Dotted lines indicate the threshold for statistical significance. For the majority of neurons in both the model and experimental data (See Fig. 3A in [5]), the attentional effect on contrast-response functions is significantly better explained by response gain.

Figure 9 shows a scatter plot of the Z-transformed correlations for the contrast-gain and response-gain models from the simulation data. Filled circles represent neurons that were significantly better fit by the response-gain model, and open circles are neurons for which neither model provided a statistically significantly better fit. Dotted lines mark the statistical criterion of 1.645, corresponding to a 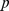-value of 0.05.

Of the 200 model neurons, only one could not be distinguished as being better fit by either model, with the remaining 199 being significantly better explained by response gain (Figure 9). As with the simulation data from the ARC, the vast majority of cells in the experimental data (Fig. 3A in [5]) were better explained by a response gain.

The simulations demonstrate that, were the data only assessed using a contrast gain model, it could be concluded that contrast gain provides an excellent explanation of the phenomena. However, closer inspection of the data by quantitatively comparing both contrast-gain and response-gain models, reveals that the data are significantly better explained by a response-gain model.

As with the results of the previous two simulations, the distribution of the simulation data was significantly narrower than the experimental data, despite a similar number of repetitions being performed (the experiment performed 36 trials on average, and the simulations used exactly 36 trials), and the same analysis methods being used. Qualitatively consistent results were obtained without the inclusion of noise at the input level, although there was significantly less variability across repetitions. Several possible sources of trial to trial variability may have influenced the experimental recordings that are not present in the model, such as measurement error, stimulus processing effects, changes in the animal's cognitive state and alertness, and involvement of different cell assemblies. Given the paucity of data that can inform the selection of the most appropriate method for modelling this variability, the simple approach used here provides a suitable approximation.

## Discussion

The ARC was used to simulate three experiments of attentional processing. Because the model is fully spiking, with each simulation, the model data was analyzed using the same methods as in the original experiment, and was shown to be quantitatively consistent with the experimental results. We now discuss how the ARC's mechanism for attentional routing provides a unifying explanation for three seemingly different forms of attentional modulation.

In Womelsdorf et al. [3], it was demonstrated that spatial attention alters the spatial width and position of cortical RFs, but not the gain. By and large, our results are also consistent with those of Anton-Erxleben et al. [44], which reinforces several of the findings reported in Womelsdorf et al. [3]. Specifically, as with Womelsdorf et al. and ARC, Anton-Erxleben et al. report changes in the size and width, but not amplitude of the classical receptive field (cRF; the excitatory centre of the receptive field) with shifts of spatial attention. However, whereas Womelsdorf et al. [3] and ARC only consider the cRF, Anton-Erxleben et al. further examine the changes in the RF surround, finding a non-multiplicative push-pull modulation of the receptive field's center-surround. To model this study using ARC, the feedforward connection weights would need to be extended from the presently used Gaussian connectivity pattern, to a difference of Gaussians (DoG) that better captures the RF surround, beyond the cRF we have used here. This extension would also better align ARC for simulations of a recent study by Niebergall et al. [45], which found similar changes in RF sizes with attention. While it remains to be demonstrated that utilizing a DoG connectivity profile would allow ARC to reproduce Niebergall et al.'s finding of a seemingly discrete and divided attentional focus, we believe that, with the above extension, ARC would be consistent with their reports of an RF expansion with shifts of spatial attention.

Both Treue and Martinez-Trujillo [2] and Lee and Maunsell [5] show that spatial attention imposes a multiplicative scaling of responses across all stimulus values, without affecting selectivity. In Treue and Martinez-Trujillo [2], this effect was seen as an increase in tuning curve gain without sharpening, and in Lee and Maunsell [5], as a response gain across all contrast values, without a disproportionate increase of responses to lower contrasts. That is, in both studies, neuronal responses were similarly scaled for all stimulus values, without a preferential scaling of values distant from the peak of the tuning curve.

We note that a subsequent study by Martinez-Trujillo and Treue [46] that examined feature-based attention rather than spatial attention, found that the tuning curves for motion direction exhibited sharpening as a function of the motion direction being attended. The ARC suggests that this sharpening in feature-based attention is equivalent to the sharpening (i.e. shrinking) of spatial receptive fields in spatial attention. As such, in the Lee and Maunsell [5] study of spatial attention, the absence of changes in tuning curve width for contrast (i.e. response gain), is consistent with our proposal that the sharpening of tuning curves occurs for the feature being attended, be it motion, contrast, or the spatial dimension.

Many studies of attentional effects on contrast response functions have been conducted, and now we discuss a few of the more prominent studies showing contrast-gain. A recent study by Khayat et al. [47] measured responses in MT in a feature- and spatial-based attention task, and examined whether the data were better explained by a contrast or response gain models, concluding that contrast gain better fits the data. However, that study required the animals to shift both feature- and spatial-based attention, and as noted by Herrmann et al. [48], the interaction of attention signals may have contributed to the response increase when attending fixation and suppressed responses when attending to the distant moving stimulus pair.

An earlier study by Martinez-Trujillo and Treue [41] reported attentional modulation in MT cells that is consistent with contrast-gain. However, this study presented each stimulus for 1000 ms and analysed the average activity from 200–1000 ms following stimulus onset. Such long stimulus presentations may provide sufficient time for adaptation to low contrast stimuli, which may also obscure this effect. Although [41] reports a contrast gain effect, response gain was also found to provide a good fit to the data, as the correlation coefficients for both models were greater than 0.82 across neurons.

An early study by Reynolds et al. [40] also reported contrast gain between attend-out and attend-in conditions with a single receptive field stimulus, although that study did not quantitatively test whether the data were better fit by a response gain or contrast gain model. As noted by Lee and Maunsell [5], only a small number of cells in the Reynolds et al. study could not be explained by response gain; rather, the data reported by Reynolds et al. suggest that, were a quantitative analysis performed, the response gain model would also provide a suitable description of the modulation effect.

In addition to the stimulus and task design, the discrepancies between these studies may arise from the different analysis methods being used. Reynolds et al. [40], as well as a study in superior colliculus by Li and Basso [49], both report attentional effects that were consistent with contrast gain. However, both analyzed examined neural responses using a receiver operator characteristic (ROC), which compresses differences at higher contrast values, where the effects of response gain are most evident [42]. Using a similar experimental method to that of Reynolds et al. [40], Williford and Maunsell [42] found that most of the attentional effects could be marginally better explained by response gain or activity gain than by contrast gain.

However, using a single model, with only 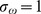 and 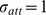 set as parameters, these seemingly different forms of attentional modulation were reproduced by the ARC using the same mechanisms. This kind of unification, particularly in a detailed spiking implementation as provided by the ARC, is presently not found in other attention models.

### Comparison to Other Models

Related to the ARC is the gain field model by Salinas and Abbott [7], which also employs a Gaussian gain field to modulate feedforward visual signals. While their model was shown to be qualitatively consistent with reports of RF shifts, it is not implemented in spiking neurons, is not defined for an entire cortical area or more than two layers of connectivity, and has not been quantitatively compared to specific physiological results. Consequently, this model has not been shown to provide a mechanism that can explain the diversity of results captured by the ARC.

The ARC is closely related to the shifter circuit [6], which provides a high level account of selective routing throughout the visual hierarchy. The ARC incorporates several elements of the shifter circuit, but addresses some of its limitations, namely: 1) its non-spiking implementation that precludes making quantitative comparisons to physiology studies; 2) its requirement of highly specific long range pulvinar projections for transmitting local control signals; and 3) its reliance on fast synaptic weight changes to perform selective routing. Specifically, the ARC is a spiking model, and does not share assumptions 2) and 3) with the shifter circuit. With regard to assumption 2) in particular, while ARC does not require control signals with the specificity of those used in the shifter circuit, it does require a structured control signal input. The primary differences are that in ARC, a single, and comparably simple global control signal (i.e. target size and position) exists for a given attentional target and is utilized across cortical areas. For the purposes of the studies modelled here, we found that 100 neurons were sufficient to encode and transmit these control signals with suitable precision. With regard to its application to the dorsal stream, ARC only requires that the global control signal be broadly distributed to the top level of the visual hierarchy, such as PIT, or dorsally to VIP/LIP. Neurons in VIP/LIP have suitable connectivity with pulvinar, sufficiently large RFs that are capable of encoding visual information from a large portion of the visual field, and can propagate the control signals downward through the hierarchy [16], [50]

ARC is also distinct from other non-spiking models, including SAIM [19], Compte and Wang [9], Montijn et. al [51], biased competition [8] and normalization models [10] that cannot be directly quantitatively compared to physiology data. More critically, the biased competition and normalization models predict that in the Treue and Martinez-Trujillo [2] experiment, directing spatial attention to a preferred RF stimulus will produce a sharpening of non-spatial tuning curves for motion direction. However, this is in direct conflict with the experimental data of Treue and Martinez-Trujillo [2] and simulation data from ARC. Reynolds and Heeger [10] suggest that this experimental result is not inconsistent with the model, as a later study by Martinez-Trujillo and Treue [46] demonstrated tuning curve sharpening. Crucially, the later study by Martinez-Trujillo and Treue specifically examined feature-based attention rather than spatial attention, and thus the results of these two studies are not necessarily "in conflict'' with each other. Rather, the earlier study clearly demonstrates that spatial attention does not affect the selectivity for motion direction (i.e. the cells exhibit response gain but not tuning curve sharpening), whereas the later study concludes that attending to motion direction does affect the selectivity for motion direction.

Further, ARC is distinguished from the normalization model, as the latter can use highly focused and spatially narrow local modulations. Simulations using spiking neurons in the ARC found that while the ensembles of cells in layer-IV are able to perform accurate routing with wider routing functions, they had limited ability to compute and apply narrow routing functions. Specifically, early experimentation using a Laplace routing function rather than a Gaussian (Equation 7) revealed that the small populations of cells were unable to approximate the function with sufficient accuracy. Similar effects are anticipated with very small values of 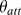 in the current model, and merit further investigation. However, it remains to be shown that, were the normalization model to be implemented in spiking neurons, the model could accurately compute the such narrow functions within biological constraints.

### Predictions

The most direct, quantitative prediction of the model is that the narrower confidence intervals in Figures 6 and 7 reflect how these distributions would change with additional experimental trials. The smoother and narrower distributions of data in the simulations result from the recording of significantly more neurons than the original experiments, with 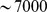 simulated neurons versus 78 in Womelsdorf et al. [3], 8500 versus 56 in Treue and Martinez-Trujillo [2], and 200 versus 56 in Lee and Maunsell [5].

A second prediction of the model is that the effects of spatial attentional on contrast response functions are independent of target size; that is, the size of the target will not cause cells to differentially exhibit response gain or contrast gain effects. The ARC's mechanisms for selective routing predict that spatial attention only affects neural spatial selectivity, without altering selectivity for other feature dimensions, a prediction that distinguishes the ARC from the normalization model [10].

More qualitatively, the primary prediction of the ARC is that the seemingly different forms of attentional modulation are consequences of a single mechanism for selective attentional routing. Specifically, the model suggests that attending a particular feature dimension imposes a Gaussian shaped multiplicative gain term defined over the dimension being attended, and centred on the target feature. In the case of spatial attention, the gain term is defined spatially, and centred on the target's RF position. This same mechanism is equally well-defined over any other feature dimension. Although not explored in detail here, this is consistent with the observation of tuning curve sharpening with feature-based attention [46].

A fourth qualitative prediction relates to the timing of attentional modulation in different cortical areas, where higher cortical levels are affected earlier than lower levels [4], [52], [53]. The ARC proposes that this effect results from attentional control signals being fed back through the visual hierarchy, where they influence neuronal activity at each area in turn (see Figure 1). As the control signals are computed, applied and fed back at each level, the effects of attention will be observed propagating back through the visual hierarchy. Depending on the amount of spatial detail required, successful recognition of the target may be performed without needing to propagate the control signal down through the entire hierarchy, which may explain the lack of attentional effects being observed in lower areas when simple tasks are used [1], [38], [39].

A fifth prediction comes from consideration of the 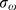 parameter in the model. In each simulation, for simplicity, the connection strengths across the RFs had a Gaussian profile with 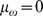 and 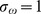. If a smaller value for 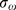 were used instead, the inputs at the RF edges would be more strongly attenuated. With a smaller value for 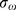 in the Womelsdorf et al. experiment, the model predicts that directing attention to a stimulus at the RF edge will result in a decreased amplitude of the RF fits, and thus a reduction in gain. A more accurate estimate of 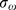 in ARC could be established by repeating the Womelsdorf et al. experiment and systematically shifting the target positions toward the RF edge. If the RF gain is reduced as a function of target position, the amounts by which the RF size changes can be used to construct an estimate for 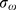.

The sixth qualitative observation from the model, for which there is some support already [4], [53], is that an increasing proportion of neurons at each higher cortical level will show attentional modulation, as a result of neurons in these areas switching from their default routing state to a selective routing state during attentionally demanding tasks. The ARC further predicts that the magnitude of modulation will increase in higher cortical areas.

A final observation of model performance, is that neurons operate in a "default'' routing state, both prior to the target being known, and when they are not encoding information from the target (i.e., when they are not tuned to the features being attended, or when their RF does not contain the target). This aspect of the model can be tested by evaluating the following three predictions: 1) default state neurons will on average have a larger RF when their RF does not contain a target; 2) in the Womelsdorf task, neurons with peripheral RFs will on average respond more strongly to probe stimuli on the side of their RF that is more distant from the fovea; and 3) in the Womelsdorf task, when two stimuli are presented in the RF, there will be a larger shift on average when attention is directed to the target closer to the fovea than when attending the other RF target.

While these predictions and observations are of varying degrees of specificity, they are each empirically testable. The most clearly testable are those suggesting specific distributions of neuron response functions, which are direct extensions of the experiments reproduced here. As well, observations five and seven can be tested without varying the setup of the Womelsdorf et al. task, and so are perhaps the best combination of novel and practical. Predictions four and six can be tested by simultaneously recording single cells from multiple cortical areas (e.g. V2, V4, and PIT) while the animal performs a spatial attention task, and measuring the proportion of cells in each area showing modulation, the strength of modulation, and the temporal onset of attentional effects. Predictions two and five are clearly more qualitative and so will require several different experiments to be performed.

## Conclusions

We have presented a model of visual attention that provides a mechanistic description of selective attentional processing in cortex. The model is fully implemented in spiking neurons, and the computations for performing selective attentional processing have been mapped to specific neuron types and laminar circuitry. Using this model, we simulated three studies of attention in macaque, and demonstrated that the model, without need for parameter tuning, produces quantitatively consistent results for all three studies.

The ability to capture this diverse data within a single model arises from a central tenet of the ARC, namely that the effect of spatial attention on a cell's activity is independent of the cell's preferred visual feature: pure spatial attention modulates the influence of visual inputs to a neuron based on their spatial position, and does not directly influence the cell's selectivity for other features.

It is this effect that is shown by the Womelsdorf et al. study: the spatial selectivity changes, but not the selectivity for its preferred feature. That is, the RF amplitude does not change, but the RF position and width does. This is further shown by Treue and Martinez-Trujillo [2], where the cell's feature tuning does not change (i.e. no sharpening), but rather, spatial attention modulates all feature values equally and affects their gain based on their spatial position. Finally, this same effect is demonstrated in the Lee and Maunsell study, where shifting spatial attention does not alter the cell's selectivity, as would be the case were there a contrast gain effect, but rather scales all responses based on their spatial position. We further believe that the mechanism proposed by ARC for routing information under spatial attention, can be mapped directly to feature-based attention, where attending to a given feature produces sharpening of the tuning curve around that feature value, analogous to a spatial location, but does not affect selectivity to other features.

Because ARC is defined in greater anatomical detail than many current models, it provides an opportunity to test the above proposal, as well as those listed in Predictions. As computational power increases, we envision extending the model to two spatial dimensions, using more complex stimuli, and simultaneously simulating additional hierarchical levels. As a consequence, ARC provides new opportunities for exploring the underlying basis of visual attention in a more biologically detailed, scalable and quantifiably testable model than previously available.
